# Sleep apnea in people with Down syndrome: Causes and effects of physical activity?

**DOI:** 10.3389/fneur.2023.1123624

**Published:** 2023-02-02

**Authors:** Duy-Thai Nguyen, Véronique-Aurélie Bricout, Hong-Tram Tran, Van-Hung Pham, Sy Duong-Quy

**Affiliations:** ^1^Clinical Research Committee, Vietnam Society of Sleep Medicine (VSSM), Da Lat, Vietnam; ^2^National Institute for Control of Vaccines and Biologicals, Ministry of Health, Hanoi, Vietnam; ^3^University Grenoble Alpes, Inserm U1300, CHU Grenoble Alpes, HP2, Grenoble, France; ^4^Sleep Lab Centre, Bio-Medical Research Centre, Lam Dong Medical College, Da Lat, Vietnam; ^5^Immuno-Allergology Division, Hershey Medical Center, Penn State Medical College, Hershey, PA, United States; ^6^Sleep Lab Unit, Outpatient Department, Pham Ngoc Thach Medical University, Ho Chi Minh City, Vietnam; ^7^Department of Respiratory Functional Exploration, University Medical Center, University of Medicine and Pharmacy, Ho Chi Minh City, Vietnam

**Keywords:** sleep disorders, sleep apnea, physical activity, Down syndrome, trisomy 21

## Abstract

Poor sleep quality is recognized as a major risk factor for poor health, increasing the incidence of serious chronic diseases. In people with Down syndrome, sleep apnea prevalence is significantly greater, it is caused by genetic, anatomical, endocrine, and metabolic abnormalities. The consequences of sleep disruption due to sleep apnea are very serious, especially in terms of neurocognitive and cardiovascular effects, leading to reduced life expectancy and quality of life in this population. However, the management, care, and treatment of related disorders in people with Down syndrome are still inadequate and limited. Therefore, this article wants to increase understanding and awareness about sleep apnea and the benefits of physical activity in improving sleep quality in the Down syndrome community, families, and their care specialists.

## 1. Introduction

Sleep is a biological process essential to life. It plays a fundamental role in the functioning of the brain, metabolism, systemic functions, regulation of appetite, but also in the regulation of the immune, hormonal and cardiovascular systems (1). Besides it, sleep is essential for recovery and good mental health. Healthy, normal, good-quality sleep is characterized by duration, regularity, absence of sleep disturbances and/or decreased wakefulness hence decreased daytime sleepiness (2, 3). Sleep is also an essential element for learning, memory, emotional control, and mood maintenance. However, today, a large part of the population “sleeps badly” and goes to bed later and later, thus reducing the amount of sleep. The abuse of screens, work pressure or certain professional specificities requiring night work (sleep deprivation) affect sleep and ultimately health and quality of life (4). The decline in sleep quality in children, adolescents and adults is a real problem in our society because sleeping poorly is reported to affect approximately one-third of the general population (5). Its consequences lead to impaired cognition, lower quality daily activities, poorer work performance, and impaired physical and mental health (6, 7).

From clinicians who consider the increasingly frequent complaints related to an alteration of sleep quality to the large industrial companies who market more and more devices allowing the detection of sleep abnormalities and the quantification of their duration in outpatients, it can be seen that many individuals and organizations were indeed interested in this issue.

Actually, sleep is an essential time for the recovery and homeostasis of physiological processes. It is not a lost time but a time during which cerebral activities allow the processing of acquisitions, the consolidation of learning and their memorization. Controlled by two major factors, homeostatic factors, and circadian factors (biological clock), sleep is also strongly influenced by many other factors (noise, state of health, stress, light, temperature...). Therefore, any quantitative and/or qualitative alteration of sleep has an impact on the daily performance, and increases many risks, in particular those related to health: obesity, diabetes, cardiovascular diseases, decrease in immunity, increase in mortality and ultimately poor quality of life.

Today, variations in many environmental and social conditions, have caused sleep disorders to become common and frequent (8, 9), but their management and treatment have long been underestimated. Compared to other health issues, sleep health is a topic that has only recently been studied (10), especially for vulnerable individuals such as those with disabilities.

People with disabilities have physical, motor, or intellectual specificities that can cause certain sleep disorders (such as breathing disorders) and affect their quality of life (11). According to the World Health Organization, up to 15% of the world's population has a specific form of disability with associated sleep disorders (12). For example, several studies have shown that 86% of children and adults with disabilities had symptoms related to sleep disorders (sleep apnea syndrome, narcolepsy...) resulting in sleep deprivation, and daytime sleepiness... (13). The health consequences for populations with disabilities are multiple (14–16). Sleep disorders, if left untreated, can progress to serious chronic diseases that increase the risk of death and decrease life expectancy in people with disabilities (17).

Down syndromes (DS) represent a significant population in the disability community. They are characterized by a high prevalence of sleep problems, such as sleep apnea syndrome, which manifest from early childhood through late life. Sleep apnea syndrome (SAS) in people with DS is characterized by frequent pauses in breathing during sleep, causing oxygen desaturation and arousals (18). Hypoxemia due to these apnea episodes can occur several hundred times during the night, increasing the risk of cardiovascular complications and hypertension in these subjects (19). In addition, dysfunctions of physiological functions and anatomical abnormalities in people with DS facilitate overexpression of SAS and exacerbate acquired pathologies in this population (20). Studies on the treatment and management of sleep disorders in people with disabilities, particularly with Down syndrome, show several options. One of the most frequently used treatments for sleep disorders is the prescription of drug treatments, which are certainly effective, but which have many potential side effects. In people with Down syndrome, the therapeutic option is more complex because of the cognitive handicap, the anatomical malformations, and the frequent non-observance of the proposed treatments. Therefore, understanding the etiology and characteristics of specific sleep disorders in this population is extremely important to find more effective treatments.

## 2. Characteristics of sleep disorders in people with Down syndrome

### 2.1. Historical background and definition

Trisomy 21 (T21), or Down syndrome, was initially described in 1838 by the French physician Jean-Etienne Esquirol, and in 1846, Dr. Edouard Seguin, proposed a clinical description of Trisomy 21 based in part on the writings of Jean-Etienne Esquirol (21). In 1866, the English physician John Langdon Down published the article “Observations on an ethnic classification of idiots” in the London Hospital Reports (22) and gave the name “Mongolian type” to the profile of T21 children. He observed that this handicap always appeared congenitally, and never after birth.

On January 26, 1959, the French doctors Jérôme Lejeune, Marthe Gautier and Raymond Turpin published an article in the Bulletins de l'Académie des Sciences (23) which was to become a milestone in the treatment of children with trisomy 21. They presented the cases of three children with Down syndrome and demonstrated that this syndrome is caused by the presence of a supernumerary chromosome 21. Down syndrome was then renamed “trisomy 21.”

Trisomy 21 is the first chromosomal anomaly described in humans. It is also the first syndrome for which a link between genotype and phenotype has been established.

Trisomy 21 is defined not as a disease but as a syndrome. It is a genetic syndrome, due to the presence of a supernumerary chromosome 21. A person with Down syndrome can live in good health, in a complete state of physical, mental and social wellbeing. It only requires adaptations to his condition concerning his physical environment, his social environment, his organization, language etc.

### 2.2. Epidemiology and etiology

**Figure 1:**
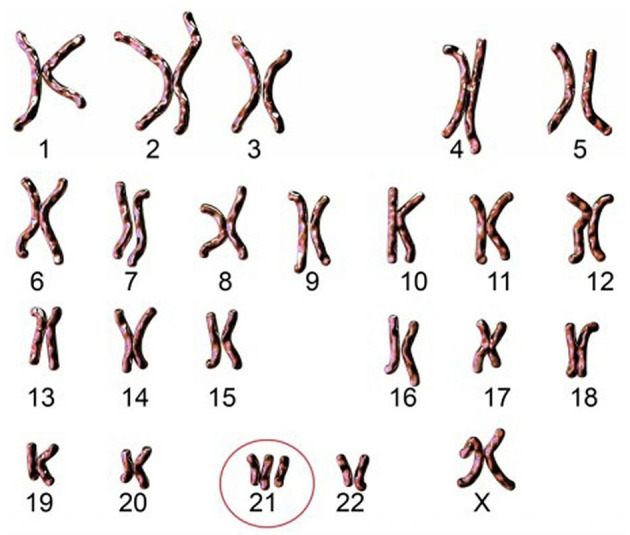
Karyotype of an individual with free and homogeneous trisomy. Source: Imago/Science Photo Library.

Trisomy 21 is the most common chromosomal anomaly, and it manifests itself from the beginning of pregnancy (Figure 1). Its prevalence is 1 in 700 births, which represents about 1,100 new cases per year in France, for a total population of about 60,000 people with T21. In France, the number of births of children with T21 is decreasing thanks to efficient screening and prenatal diagnosis. However, the risk of giving birth to a child with T21 increases with the mother's age. Thus, at 20 years of age, the risk of having a pregnancy with a trisomy is 1/2,000, whereas this risk increases to 1/20 at 45 years of age.

Today, it is reported in France one birth of T21 child per day, despite this very well-organized antenatal screening. This screening includes numerous examinations during the pregnancy (questioning, ultrasound, and biological examinations), and especially in case of doubt, a screening with specific serum markers. Thanks to these examinations, a risk calculation is obtained, and it is then possible to perform a fetal karyotype to establish the type of T21. This karyotype makes it possible to identify the precise type of T21 among the three known forms which are free and homogeneous trisomy, mosaic trisomy and trisomy by translocation. Each of these three forms is distinguished by different clinical features. All of them are the result of an abnormality brought on by chromosome 21.

This chromosome 21 alone expresses 1.5% of the human genome, the information is thus carried on 47 million nucleotides representing 300–400 genes that code for different proteins. The genetic importance of chromosome 21 explains why this gain of genetic material leads to a significant biochemical imbalance with multiple and irreversible consequences.

### 2.3. Risk factors and sleep apnea

Children with Down syndrome present clinical signs whose expression and severity vary according to the type of Down syndrome. However, according to the proposed treatments, thanks to early diagnosis, thanks to physical activity and/or cultural activities programs... the life expectancy of these children has increased considerably over the last decades.

However, this improvement is based on a heavy and constraining medical course for the child and his parents, because trisomy 21 predisposes to hormonal, cardiac and metabolic dysfunctions, numerous and sometimes severe. It is associated with a wide spectrum of cognitive deficiencies (learning, memory, and language), delays in motor and neurological development (24, 25). Structural abnormalities of the brain (reduced brain volume, especially at the frontal, temporal and cerebellum levels) are predictive of dementia, and signs of Alzheimer's disease are much more frequently described in the population with T21. These abnormalities are known to be the cause of poor cognitive activity and rapid decline (24, 26).

The severity and frequency of clinical signs vary, but almost all are related to the development of sleep disorders that are more common in this population. In 2020, Dumortier and Bricout (19) published a review of the literature regarding the presentation of these links between T21 and sleep apnea syndrome. The authors of this review highlighted the causes and consequences between T21 and OSAS, specifying their birelation (OSAS in adult with Down syndrome: causes and consequences: Is it a “chicken and egg” question?).

Other studies showed the physical phenotype of T21 is very characteristic:

- The face is round, flat, and with little relief,
- The skull is small and the occiput flat,
- The eyes are oblique, spaced, and the eyelid is without fold,
- The ears are round and small,
- The nose is small,
- The tongue is very particular: thick, and rough, with many folds (macroglossia),
- The mouth presents anatomical anomalies, small size, dental problems, and prognathism.

In addition, there are anatomical vertebral particularities (instability of the first two cervical vertebrae, prevalence of narrow lumbar canal) that induce pain during activities (walking, shift work or physical activities). Both children and adults are small in stature and are characterized by overweight or obesity.

Associated with these specific physical characteristics, other clinical signs have been reported (endocrine, metabolic, autonomic). In this manuscript, we will limit ourselves to those that have a direct impact on sleep disorders.

#### 2.3.1. Endocrine disorders

Endocrine disruption has been reported in individuals with DS and it is strongly linked to obesity due to dysautonomia and the increase in fat mass, thereby indirectly causing SAS. Abnormal levels of hormones involved in metabolism, storage and lipolysis, such as leptin and insulin, have been observed to increase the risk of sleep apnea and cardiovascular complications (27). The best-described endocrine pathologies in Down syndrome are thyroid dysfunctions (hypothyroidism, hyperthyroidism) (28, 29). Hypothyroidism has major effects on basal metabolic rate and is thought to promote overweight. Hyperthyroidism induces tachycardia with palpitations and is very frequently diagnosed in conjunction with sleep disorders. These thyroid dysfunctions have multiple consequences: slowed cognitive development, fatigability, lowered energy metabolism, slowed development and growth.

Abnormalities in catecholamine secretion are also reported, with a decreased amplitude of adrenergic responses that blunt cardiovascular responses during stress or exercise (30, 31).

Furthermore, in men with T21, a decrease in testosterone secretion associated with hypogonadism is described. This acts directly on body composition, favoring the accumulation of fat mass (32). This development of fat mass has many consequences on the appearance of obstructive apneas by an accumulation effect in the upper airways.

#### 2.3.2. Metabolic dysfunctions

A sedentary lifestyle is the source of the metabolic syndromes associated with obesity, low-grade inflammation, and diabetes in people with DS. It maintains a loop of “ataxia—overweight—sedentary” and results in lower muscle mass, increased fat mass and finally SAS (33). Studies have shown that hypoxia caused by SAS induces inflammatory cytokine release leading to dysglycemia and insulin resistance in the T21 population. Meanwhile, insulin resistance also increases the risk of diabetes and obesity, which contributes to SAS (34).

In addition, generalized hypotonia increases the risk of airway collapse and airway obstruction (complete or partial) can occur during sleep, due to decreased muscle tone in the oropharynx, leading to snoring and OSA in individuals with DS (35). It also alters the quality of motor skills and effort in people with T21 (36–39). These impairments make activities of daily living more taxing and difficult to continue, with fatigability occurring more rapidly during exertion. Spontaneously, people with T21 are less inclined to engage in physical activities, and they then fall into a loop of deconditioning, sedentary lifestyle, weight, fatigue and activity limitation. Numerous studies have confirmed this vicious circle of deconditioning, which results in a lower tolerance to effort in people with T21 (36, 40).

In T21, there are more frequent digestive dysfunctions and also contribute to the development of metabolic syndrome, in particular the presence of gastroesophageal reflux (41). There is more evidence for a link between SAS and gastroesophageal reflux disease. In which, the large negative intrapleural pressure fluctuations during apnea will cause reflux phenomena (42). This may also be involved in the reduced desire to practice physical activity, as the subjects describe more reflux during exercise. These digestive disorders are also particularly observed when there is low-grade inflammation, with high inflammatory markers (43), or dysautonomia, with an altered vagal tone favoring gastroesophageal reflux.

#### 2.3.3. Autonomic nervous system dysfunctions or dysautonomias

Nervous system dysfunction has been the subject of numerous studies in the T21 population, it often presents with dysautonomia at rest, during sleep, during exercise, or during stimulation tests with an inappropriate vagal response, which effectively reflects maladaptations of the autonomic nervous system dysfunctions (ANS) (44–47). Cardiovascular and neurological parameters are cyclically altered, corresponding to respiratory events due to sleep apnea syndrome at T21, particularly with low blood pressure and heart rate than in the general population. During exercise, there is joint chronotropic incompetence and poor hormonal regulation (catecholamines, cortisol, and glycemic regulation hormones) that increase the consequences of autonomic maladaptation leading to early fatigue and limitation to exercise (30, 40, 48). Hypoxia during apnea causes bradycardia, followed by tachycardia on the resumption of breathing (49). The increase in heart rate at the end of apnea may be due to catecholaminergic release and sympathetic stimulation following a respiratory event. Sleep apnea increases arterial pressures and activates nocturnal sympathetic tones that increase pulmonary arterial pressure (50). This increases the risk factors for coronary heart failure, cardiovascular death, and stroke.

One of the proposals has been to implement regular physical activity (PA) whose beneficial effects could also have various impacts: increased practice times, loss of body fat, a gain of muscle mass, a gain of strength, improvement of sleep... (51).

All of this clinical picture drawn up to characterize trisomy 21, explains to a large extent the appearance of sleep apnea syndrome in this population.

## 3. Sleep apnea syndrome and Down syndrome

In T21, sleep apnea syndrome shows an exceptionally high prevalence ranging from 40% to 88% in adults (52) and up to 97% in children (53) while only 7–13% of the general population is affected by this syndrome (54). However, sleep apnea is still misdiagnosed in T21 while it is responsible for cardiovascular pathologies and reduced life expectancy (55). The consequences of SAS can be observed in various abnormalities such as sleep fragmentation, nocturnal awakenings, snoring, morning asthenia, daytime hyper sleepiness and mood disorders.

Obstructive sleep apnea syndrome is characterized by partial or complete obstruction of the upper airway that is intermittent and repeated during sleep (18). These events are typically associated with a decrease in blood oxygen saturation and can occur several hundred times in a single night of sleep. Apnea episodes can occur during NREM or REM sleep, but are generally more deleterious to health when they occur during REM sleep (56). There are three possible types of apneas that can be observed during sleep: obstructive, central, or mixed apneas, and these hypopneas characterized by the apnea-hypopnea index (AHI), it is the total number of abnormal events measured during 1 h of sleep. To make a precise diagnosis of these sleep disorders, polysomnography (PSG) is used to calculate AHI and classified according to the following levels: AHI > 5 = mild SAS, AHI > 10 = moderate SAS, AHI > 15 = severe SAS, AHI > 30 = very severe SAS (57, 58).

While PSG is indeed the gold standard for diagnosing SAS, there are still other warning signs that exist. Nocturnal events such as difficulty in falling asleep, multiple awakenings, sleep fragmentation, snoring can be retained. The consequences of these poor nights are also elements of attention: fatigue, irritability, excessive daytime sleepiness, and attention and memory problems (59). However, the consequences of SAS are particularly dramatic for the cardiovascular health of patients. They must therefore be diagnosed very early in order to be managed rapidly to avoid the development of pathologies that are deleterious to health and quality of life.

The diagnosis of SAS can only be established after a rigorous clinical investigation, based on several diagnostic elements (questionnaires, polysomnography…). Treatment then usually consists of the use of a continuous positive airway pressure (CPAP) machine during sleep (60). This treatment is sometimes very restrictive for the patient and therefore poorly tolerated. Today, one of the proposals made in T21, when the AHI is not severe and the patient refuses to use devices at night, is based on a physical activity program associated with the implementation of strict hygienic and dietary measures.

Interest in the effects of physical activity for populations with cognitive disabilities is a relatively recent topic. Physical activity has been proposed for about a decade as a non-pharmacological interventional method that has many benefits for children with disabilities and sleep disorders. However, offering physical activity to these populations must also be done within a rigorous and safe framework in order not to induce additional deleterious effects.

## 4. Physical activity and Down syndrome

**Figure 2:**
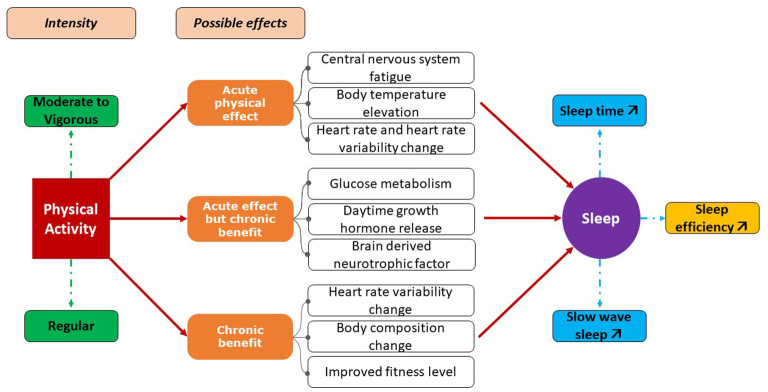
Positive effects of physical activity on sleep.

Physical Activity is defined as the movement of the human body that requires energy expenditure (61). It is considered an effective, non-pharmacological approach to improving sleep. The scientific literature on the links between sleep and physical activity is now particularly rich and the consensus on the bi-directional links between sleep and physical activity is also well demonstrated (Figure 2). Healthy sleep of sufficient quality and quantity contributes to better physical performance and conversely regular physical activity improves sleep (62, 63). However, physical activity is considered to have positive effects on sleep quality if indeed the adjustment of factors such as level, intensity and duration of exercise is adequate (64). For example, significant improvements in sleep efficiency, sleep latency, sleep duration, and nocturnal wakefulness times are reported when physical activity is increased and controlled (64).

The benefit of PA in the management of SAS for the population with Down syndrome has been demonstrated (65, 66). PA not only reduces the severity of SAS but also improves autonomic function through decreased sympathetic tone (67). Some studies have also reported positive effects of regular physical activity on physiological responses to stress (38), weight loss, and cardiovascular responses to exercise (68–70).

However, some anatomical peculiarities of T21, described previously, may limit the ability to perform certain types of effort, and at the same time explain the prevalence of SAS in this population. Many malformations (macroglossia, glossoptosis, narrow upper airways) that are well described in the responsibility of obstructive apneas, limit ventilatory exchange during physical activity (71).

Although PA has clear benefits in limiting the health consequences of SAS in T21 and non-T21 patients with Down syndrome, there are a variety of factors that not only contribute to worsening the consequences of SAS, but also act as barriers to the management of these sleep disorders.

The practice of PA is not yet offered in all establishments that receive people with Down syndrome. The institutionalization of people with T21 and their drug treatments are factors that limit physical activity.

The cognitive characteristics of T21 (early aging, intellectual deficit, difficulties in understanding instructions, low motivation to practice) also constitute barriers to physical activity.

However, encouraging the practice of a regular physical activity is a major challenge. In this sense, several studies have shown that for young people with T21, group activities should be preferred (72, 73) which are more in line with the social character of people with T21. Several studies have shown the benefit of re-training programs in T21 with an improvement in physical fitness with benefits on aerobic capacity (74), cardiovascular function (69, 75), muscular strength, balance and motor function (76–80). Improving this fitness simultaneously contributes to a decrease in body fat (76–80).

Finally, the benefits of PA on cognitive abilities in individuals with T21 have also been widely described (81). The management of sleep apnea through physical activity in T21, helps to combat a sedentary lifestyle and reduce the risk factors associated with SAS (82). It also increases sleep duration and quality (77, 83–85). In order to improve the health and quality of life of individuals with T21, early interventions and physical activity recommendations should be offered to value a healthy lifestyle and improve understanding of the benefits on sleep quality and quantity.

## 5. Conclusion

Individuals with Down syndrome had adverse genetic, anatomical characteristics that increase the risk of sleep apnea and mobility limitations, meanwhile physical activity has been reported with positive effects on sleep quality through a significant reduction in the apnea-hypopnea index in this population in this population. However, we need to understand more about the relationship between quantity and level of physical activity on sleep quality to provide information and recommendations for the design the optimal physical activity programs for the Down syndrome community, thereby helping to improve their quality of life.

## Author contributions

D-TN drafted the initial manuscript and revised the final manuscript. V-HP support to improve the manuscript after the review process. V-AB, H-TT, and SD-Q revised the manuscript. All authors approved the final manuscript as submitted and agreed to be accountable for all aspects of the work.

## References

[1] Jee HJ Shin W Jung HJ Kim B Lee BK Jung Y-S. Impact of sleep disorder as a risk factor for dementia in men and women. Biomol Ther (Seoul). (2020) 28:58–73. 10.4062/biomolther.2019.192 31838834 PMC6939686

[2] Malhotra P. Sleep, its attributes, deprivation and hygiene: a recapitulation. Natl J Commun Med. (2019) 10:678–83. Available online at: https://njcmindia.com/index.php/file/article/view/592

[3] Watson NF Badr MS Belenky G Bliwise DL Buxton OM Buysse D. Recommended amount of sleep for a healthy adult: a joint consensus statement of the American Academy of Sleep Medicine and Sleep Research Society. Sleep. (2015) 38:843–4. 10.5665/sleep.4716 26039963 PMC4434546

[4] Jakobsson M Josefsson K Högberg K. Reasons for sleeping difficulties as perceived by adolescents: a content analysis. Scand J Caring Sci. (2020) 34:464–73. 10.1111/scs.12750 31487078 PMC7328685

[5] Grandner MA. Chapter 2 - Epidemiology of insufficient sleep and poor sleep quality. In:Grandner MA, editor. Sleep and Health. New York, NY: Academic Press (2019). p. 11–20. 10.1016/B978-0-12-815373-4.00002-2

[6] Lallukka T Sivertsen B Kronholm E Bin YS Øverland S Glozier N. Association of sleep duration and sleep quality with the physical, social, and emotional functioning among Australian adults. Sleep Health. (2018) 4:194–200. 10.1016/j.sleh.2017.11.006 29555134

[7] Hershner SD Chervin RD. Causes and consequences of sleepiness among college students. Nat Sci Sleep. (2014) 6:73. 10.2147/NSS.S62907 25018659 PMC4075951

[8] Billings ME Hale L Johnson DA. Physical and social environment relationship with sleep health and disorders. Chest. (2020) 157:1304–12. 10.1016/j.chest.2019.12.002 31870910 PMC7268445

[9] Wickwire EM Geiger-Brown J Scharf SM Drake CL. Shift work and shift work sleep disorder: clinical and organizational perspectives. Chest. (2017) 151:1156–72. 10.1016/j.chest.2016.12.007 28012806 PMC6859247

[10] Knowlden AP Sharma M Bernard AL A. theory of planned behavior research model for predicting the sleep intentions and behaviors of undergraduate college students. J Prim Prev. (2012) 33:19–31. 10.1007/s10935-012-0263-2 22293980

[11] Turk J. Sleep disorders in children and adolescents with learning disabilities and their management. Adv Mental Health Learn Disabil. (2010) 4:50–9. 10.5042/amhld.2010.0059

[12] Bright T Wallace S Kuper H. A systematic review of access to rehabilitation for people with disabilities in low-and middle-income countries. Int J Environ Res Public Health. (2018) 15:2165. 10.3390/ijerph15102165 30279358 PMC6210163

[13] Bruni O Novelli L. Sleep disorders in children. BMJ Clin Evid. (2010) 9:2304. PMC3217667 21418676

[14] Richdale AL Baker EK. Sleep in individuals with an intellectual or developmental disability: recent research reports. Curr Dev Disord Rep. (2014) 1:74–85. 10.1007/s40474-014-0010-x

[15] Guarnieri B Adorni F Musicco M Appollonio I Bonanni E Caffarra P. Prevalence of sleep disturbances in mild cognitive impairment and dementing disorders: a multicenter Italian clinical cross-sectional study on 431 patients. Dement Geriatr Cogn Disord. (2012) 33:50–8. 10.1159/000335363 22415141 PMC3696366

[16] Wiggs L France K. Behavioural treatments for sleep problems in children and adolescents with physical illness, psychological problems or intellectual disabilities. Sleep Med Rev. (2000) 4:299–314. 10.1053/smrv.1999.0094 12531171

[17] Sharma N Lee J Youssef I Salifu MO McFarlane SI. Obesity, cardiovascular disease and sleep disorders: insights into the rising epidemic. J Sleep Disord Therapy. (2017) 6, 1000620. 10.4172/2167-0277.1000260 28638745 PMC5476303

[18] Mannarino MR Di Filippo F Pirro M. Obstructive sleep apnea syndrome. Eur J Intern Med. (2012) 23:586–93. 10.1016/j.ejim.2012.05.013 22939801

[19] Dumortier L Bricout V-A. Obstructive sleep apnea syndrome in adults with down syndrome: causes and consequences. Is it a” chicken and egg” question? Neurosci Biobehav Rev. (2020) 108:124–38. 10.1016/j.neubiorev.2019.10.018 31706958

[20] Lavrentaki A Ali A Cooper BG Tahrani AA Mechanisms Mechanisms of Endocrinology. Mechanisms of disease: the endocrinology of obstructive sleep apnoea. Eur J Endocrinol. (2019) 180:R91–R125. 10.1530/EJE-18-0411 30540561

[21] Seguin E. Traitement moral, hygiène et éducation des idiots et des autres enfants arriérés. Paris: JB Baillière (1846).

[22] Down JLH. Observations on an ethnic classification of idiots. Lond Hosp Rep. (1866) 3:259–62.

[23] Lejeune J. Etude des chromosomes somatiques de neuf enfants mongoliens. CR Acad Sci (Paris). (1959) 248:1721–2. 13639368

[24] Rachidi M Lopes C. Mental retardation and associated neurological dysfunctions in Down syndrome: a consequence of dysregulation in critical chromosome 21 genes and associated molecular pathways. Eur J Paediatr Neurol. (2008) 12:168–82. 10.1016/j.ejpn.2007.08.010 17933568

[25] Antonarakis SE Lyle R Dermitzakis ET Reymond A Deutsch S. Chromosome 21 and down syndrome: from genomics to pathophysiology. Nat Rev Genet. (2004) 5:725–38. 10.1038/nrg1448 15510164

[26] Capone GT. Down syndrome: genetic insights and thoughts on early intervention. Infants Young Child. (2004) 17:45–58. 10.1097/00001163-200401000-00007

[27] Öztürk L Ünal M Tamer L Çelikoglu F. The association of the severity of obstructive sleep apnea with plasma leptin levels. Arch Otolaryngol Head Neck Surg. (2003) 129:538–40. 10.1001/archotol.129.5.538 12759266

[28] Murdoch J Ratcliffe W McLarty D Rodger J Ratcliffe J. Thyroid function in adults with Down's syndrome. J Clin Endocrinol Metab. (1977) 44:453–8. 10.1210/jcem-44-3-453 138688

[29] Tüysüz B Beker D. Thyroid dysfunction in children with Down's syndrome. Acta Paediatr. (2001) 90:1389–93. 10.1111/j.1651-2227.2001.tb01601.x 11853334

[30] Leti T Guinot M Favre-Juvin A Bricout V-A. Difference of catecholamine responses to exercise in men with trisomy 21, with or without chronotropic incompetence. Physiol Behav. (2015) 142:97–103. 10.1016/j.physbeh.2015.02.007 25660758

[31] Leti T Guinot M Favre-Juvin A Pepin J-L Levy P Bricout VA. Obstructive sleep apnea syndrome in two subjects with down syndrome: continuous positive airway pressure contribution on exercise tolerance. Neurosci Med. (2012) 3:187. 10.4236/nm.2012.32024

[32] Steidle C Schwartz S Jacoby K Sebree T Smith T Bachand R. AA2500 testosterone gel normalizes androgen levels in aging males with improvements in body composition and sexual function. J Clin Endocrinol Metab. (2003) 88:2673–81. 10.1210/jc.2002-021058 12788872

[33] Rimmer JH Yamaki K. Obesity and intellectual disability. Ment Retard Dev Disabil Res Rev. (2006) 12:22–7. 10.1002/mrdd.20091 16435329

[34] Tamura A Kawano Y Watanabe T Kadota J. Relationship between the severity of obstructive sleep apnea and impaired glucose metabolism in patients with obstructive sleep apnea. Respir Med. (2008) 102:1412–6. 10.1016/j.rmed.2008.04.020 18606532

[35] Giannasi LC Dutra MT Tenguan VL Mancilha GP Silva GR Fillietaz-Bacigalupo E. Evaluation of the masticatory muscle function, physiological sleep variables, and salivary parameters after electromechanical therapeutic approaches in adult patients with Down syndrome: a randomized controlled clinical trial. Trials. (2019) 20:1–15. 10.1186/s13063-019-3300-0 30975204 PMC6460660

[36] Baynard T Pitetti KH Guerra M Unnithan VB Fernhall B. Age-related changes in aerobic capacity in individuals with mental retardation: a 20-yr review. Med Sci Sports Exerc. (2008) 40:1984–9. 10.1249/MSS.0b013e31817f19a1 18845971

[37] Fernhall B Mendonca GV Baynard T. Reduced work capacity in individuals with Down syndrome: a consequence of autonomic dysfunction? Exerc Sport Sci Rev. (2013) 41:138–47. 10.1097/JES.0b013e318292f408 23558694

[38] Fernhall B Pitetti K. Limitations to physical work capacity in individuals with mental retardation. J Clin Exerc Physiol. (2001) 3:176–85.

[39] Mendonca GV Pereira FD Fernhall B. Reduced exercise capacity in persons with Down syndrome: cause, effect, and management. Ther Clin Risk Manag. (2010) 6:601. 10.2147/TCRM.S10235 21206759 PMC3012449

[40] Mendonca GV Pereira FD. Heart rate recovery after exercise in adults with the Down syndrome. Am J Cardiol. (2010) 105:1470–3. 10.1016/j.amjcard.2009.12.073 20451697

[41] Mitchell RB Call E Kelly J. Diagnosis and therapy for airway obstruction in children with Down syndrome. Arch Otolaryngol Head Neck Surg. (2003) 129:642–5. 10.1001/archotol.129.6.642 12810469

[42] Bermudez BE de Oliveira CM de Lima Cat MN Magdalena NI Celli A. Gastrointestinal disorders in Down syndrome. Am J Med Genet Part A. (2019) 179:1426–31. 10.1002/ajmg.a.61258 31183986

[43] Fructuoso M Rachdi L Philippe E Denis R Magnan C Le Stunff H. Increased levels of inflammatory plasma markers and obesity risk in a mouse model of Down syndrome. Free Rad Biol Med. (2018) 114:122–30. 10.1016/j.freeradbiomed.2017.09.021 28958596

[44] Fernhall B Figueroa A Collier S Baynard T Giannopoulou I Goulopoulou S. Blunted heart rate response to upright tilt in people with Down syndrome. Arch Phys Med Rehabil. (2005) 86:813–8. 10.1016/j.apmr.2004.10.027 15827937

[45] Figueroa A Collier SR Baynard T Giannopoulou I Goulopoulou S Fernhall B. Impaired vagal modulation of heart rate in individuals with Down syndrome. Clin Autonom Res. (2005) 15:45–50. 10.1007/s10286-005-0235-1 15768202

[46] Guerra M Llorens N Fernhall B. Chronotropic incompetence in persons with Down syndrome. Arch Phys Med Rehabil. (2003) 84:1604–8. 10.1053/S0003-9993(03)00342-3 14639558

[47] Heffernan KS Baynard T Goulopoulou S Giannopoulou I Collier SR Figueroa A. Baroreflex sensitivity during static exercise in individuals with Down syndrome. Med Sci Sports Exerc. (2005) 37:2026. 10.1249/01.mss.0000179217.59831.41 16331125

[48] Bricout VA Guinot M Faure P Flore P Eberhard Y Garnier P. Are hormonal responses to exercise in young men with Down's syndrome related to reduced endurance performance? J Neuroendocrinol. (2008) 20:558–65. 10.1111/j.1365-2826.2008.01695.x 18363810

[49] Guilleminault C Tilkian A Dement WC. The sleep apnea syndromes. Annu Rev Med. (1976) 27:465–84. 10.1146/annurev.me.27.020176.002341 180875

[50] Roche F. Modifications des paramètres électrocardiographiques en situation hypoxémique: applications à la physiopathologie et au diagnostic du syndrome d'apnée du sommeil. Saint-Etienne (2003).

[51] Mendonca GV Pereira FD Fernhall B. Heart rate recovery and variability following combined aerobic and resistance exercise training in adults with and without Down syndrome. Res Dev Disabil. (2013) 34:353–61. 10.1016/j.ridd.2012.08.023 23006505

[52] Trois MS Capone GT Lutz JA Melendres MC Schwartz AR Collop NA. Obstructive sleep apnea in adults with Down syndrome. J Clin Sleep Med. (2009) 5:317–23. 10.5664/jcsm.27541 19968008 PMC2725249

[53] Austeng ME Øverland B Kværner KJ Andersson E-M Axelsson S Abdelnoor M. Obstructive sleep apnea in younger school children with Down syndrome. Int J Pediatr Otorhinolaryngol. (2014) 78:1026–9. 10.1016/j.ijporl.2014.03.030 24809771

[54] Peppard PE Young T Barnet JH Palta M Hagen EW Hla KM. Increased prevalence of sleep-disordered breathing in adults. Am J Epidemiol. (2013) 177:1006–14. 10.1093/aje/kws342 23589584 PMC3639722

[55] Platt J Adeleye A Nettel-Aguirre A Chugh A Yunker W. The impact of adenotonsillectomy on obstructive sleep apnea in children with trisomy 21: a retrospective cohort study. Sleep. (2020) 43:A345. 10.1093/sleep/zsaa056.90432427548

[56] Krell SB Kapur VK. Insomnia complaints in patients evaluated for obstructive sleep apnea. Sleep Breath. (2005) 9:104–10. 10.1007/s11325-005-0026-x 16091954

[57] Ahmadi N Shapiro GK Chung SA Shapiro CM. Clinical diagnosis of sleep apnea based on single night of polysomnography vs. two nights of polysomnography. Sleep Breath. (2009) 13:221–6. 10.1007/s11325-008-0234-2 19067010

[58] Epstein L Kristo D Strollo Jr P Friedman N Malhotra A Patil S. Adult obstructive sleep apnea task force of the American Academy of sleep medicine. Clinical guideline for the evaluation, management and long-term care of obstructive sleep apnea in adults. J Clin Sleep Med. (2009) 5:263–76. 10.5664/jcsm.27497 19960649 PMC2699173

[59] Velika S Kaelan C Basir H Wuysang AD Tammasse J. Excessive daytime sleepiness due to obstructive sleep apnea. Med Clí*n Práct*. (2021) 4:100218. 10.1016/j.mcpsp.2021.100218

[60] McEvoy RD Antic NA Heeley E Luo Y Ou Q Zhang X. CPAP for prevention of cardiovascular events in obstructive sleep apnea. New Engl J Med. (2016) 375:919–31. 10.1056/NEJMoa1606599 27571048

[61] Caspersen CJ Powell KE Christenson GM. Physical activity, exercise, and physical fitness: definitions and distinctions for health-related research. Public Health Rep. (1985) 100:126. 3920711 PMC1424733

[62] Sonnentag S Venz L Casper A. Advances in recovery research: what have we learned? What should be done next? J Occup Health Psychol. (2017) 22:365. 10.1037/ocp0000079 28358572

[63] Chennaoui M Arnal PJ Sauvet F Leger D. Sleep and exercise: a reciprocal issue? Sleep Med Rev. (2015) 20:59–72. 10.1016/j.smrv.2014.06.008 25127157

[64] Healy S Haegele JA Grenier M Garcia JM. Physical activity, screen-time behavior, and obesity among 13-year olds in Ireland with and without autism spectrum disorder. J Autism Dev Disord. (2017) 47:49–57. 10.1007/s10803-016-2920-4 27671801

[65] Chen C-C Ringenbach SDR. Dose-response relationship between intensity of exercise and cognitive performance in individuals with Down syndrome: a preliminary study. J Intellect Disabil Res. (2016) 60:606–14. 10.1111/jir.12258 26923820

[66] Ng SS Chan RS Woo J Chan T-O Cheung BH Sea MM. A randomized controlled study to examine the effect of a lifestyle modification program in OSA. Chest. (2015) 148:1193–203. 10.1378/chest.14-3016 25763792

[67] Maki-Nunes C Toschi-Dias E Cepeda FX Rondon MUP Alves MJN Fraga RF. Diet and exercise improve chemoreflex sensitivity in patients with metabolic syndrome and obstructive sleep apnea. Obesity. (2015) 23:1582–90. 10.1002/oby.21126 26148219

[68] Mendonca GV Pereira FD. Influence of long-term exercise training on submaximal and peak aerobic capacity and locomotor economy in adult males with Down's syndrome. Med Sci Monitor. (2009) 15:CR33–CR9. Available online at: http://www.medscimonit.com/abstract/index/idArt/869543 19179964

[69] Rimmer JH Heller T Wang E Valerio I. Improvements in physical fitness in adults with Down syndrome. Am J Mental Retard. (2004) 109:165–74. 10.1352/0895-8017(2004)109<165:IIPFIA>2.0.CO;2 15000673

[70] Tsimaras V Giagazoglou P Fotiadou E Christoulas K Angelopoulou N. Jog-walk training in cardiorespiratory fitness of adults with Down syndrome. Perceptual Motor Skills. (2003) 96(3_suppl):1239–51. 10.2466/pms.2003.96.3c.1239 12929778

[71] Cohen WI Nadel L Madnick ME. Down Syndrome: Visions for the 21st Century. New York, NY: John Wiley & Sons (2003). 10.1002/0471227579

[72] Barr M Shields N. Identifying the barriers and facilitators to participation in physical activity for children with Down syndrome. J Intellect Disabil Res. (2011) 55:1020–33. 10.1111/j.1365-2788.2011.01425.x 21554468

[73] Menear K. Parents' perceptions of health and physical activity needs of children with Down syndrome. Downs Synd Res Pract. (2007) 12:60. 10.3104/reports.1996 17692190

[74] Ordonez FJ Rosety M Rosety-Rodriguez M. Influence of 12-week exercise training on fat mass percentage in adolescents with Down syndrome. Med Sci monitor. (2006) 12:CR416–CR9. Available online at: http://www.medscimonit.com/fulltxt.php?IDMAN=5430 17006400

[75] Mendonca GV Pereira FD Fernhall B. Effects of combined aerobic and resistance exercise training in adults with and without Down syndrome. Arch Phys Med Rehabil. (2011) 92:37–45. 10.1016/j.apmr.2010.09.015 21187203

[76] Boer P Moss S. Effect of continuous aerobic vs. interval training on selected anthropometrical, physiological and functional parameters of adults with Down syndrome. J Intellect Disabil Res. (2016) 60:322–34. 10.1111/jir.12251 26805768

[77] Chen C-CJ Spanò G Edgin JO. The impact of sleep disruption on executive function in Down syndrome. Res Dev Disabil. (2013) 34:2033–9. 10.1016/j.ridd.2013.03.009 23584183

[78] Holzapfel SD Ringenbach SD Mulvey GM Sandoval-Menendez AM Cook MR Ganger RO. Improvements in manual dexterity relate to improvements in cognitive planning after assisted cycling therapy (ACT) in adolescents with Down syndrome. Res Dev Disabil. (2015) 45:261–70. 10.1016/j.ridd.2015.08.003 26280691

[79] Jankowicz-Szymanska A Mikolajczyk E Wojtanowski W. The effect of physical training on static balance in young people with intellectual disability. Res Dev Disabil. (2012) 33:675–81. 10.1016/j.ridd.2011.11.015 22186635

[80] Li C Chen S How YM Zhang AL. Benefits of physical exercise intervention on fitness of individuals with Down syndrome: a systematic review of randomized-controlled trials. Int J Rehabil Res. (2013) 36:187–95. 10.1097/MRR.0b013e3283634e9c 23778328

[81] Vacca RA Bawari S Valenti D Tewari D Nabavi SF Shirooie S. Down syndrome: Neurobiological alterations and therapeutic targets. Neurosci Biobehav Rev. (2019) 98:234–55. 10.1016/j.neubiorev.2019.01.001 30615933

[82] Nguyen TD Baillieul S Guinot M Doutreleau S Bricout V-A. Classification of factors effect on sleep in individuals with Down syndrome. Brain Sci. (2021) 11:1500. 10.3390/brainsci11111500 34827499 PMC8615686

[83] Chen C-C Ringenbach SDR. Walking performance in adolescents and young adults with Down syndrome: the role of obesity and sleep problems. J Intellect Disabil Res. (2018) 62:339–48. 10.1111/jir.12474 29484778

[84] Tracey KJ. Physiology and immunology of the cholinergic antiinflammatory pathway. J Clin Invest. (2007) 117:289–96. 10.1172/JCI30555 17273548 PMC1783813

[85] Matsumoto T Miyawaki T Ue H Kanda T Zenji C Moritani T. Autonomic responsiveness to acute cold exposure in obese and non-obese young women. Int J Obes. (1999) 23:793–800. 10.1038/sj.ijo.0800928 10490779

